# Cyclodextrin-assisted synthesis of tailored mesoporous silica nanoparticles

**DOI:** 10.3762/bjnano.9.64

**Published:** 2018-02-22

**Authors:** Fuat Topuz, Tamer Uyar

**Affiliations:** 1 UNAM-National Nanotechnology Research Center, Bilkent University, 06800 Ankara, Turkey; 2 Faculty of Engineering and Natural Sciences, Sabanci University, 34956 Istanbul, Turkey; 3 Institute of Materials Science and Nanotechnology, Bilkent University, 06800 Ankara, Turkey

**Keywords:** cyclodextrin, faceted particles, mesoporous silica nanoparticles (MSN), microporous

## Abstract

Mesoporous silica nanoparticles (MSNs) have sparked considerable interest in drug/gene delivery, catalysis, adsorption, separation, sensing, antireflection coatings and bioimaging because of their tunable structural properties. The shape, size and pore structure of MSNs are greatly influenced by the type of additives used, e.g., solvent and pore-templating agent. Here, we studied the influence of cyclodextrin (CD) molecules on the formation of MSNs. The nanoparticles over 100 nm in diameter were synthesized by surfactant-templated, hydrolysis–polycondensation reactions in the presence of pristine CD (β-CD) or hydroxypropyl-functionalized CDs (HP-γ-CD and HP-β-CD). Depending on the formulation conditions, differently shaped MSNs, such as bean-like, spherical, ellipsoid, aggregate and faceted were generated. The morphology and size of MSNs varied with the CD-type used. Generally, spherical particles were obtained with β-CD, while a faceted morphology was observed for the particles synthesized using HP-CDs. The particle size could be tuned by adjusting the amount of CD used; increasing the CD concentration led to larger particles. MSNs synthesized in the presence of β-CD displayed a smaller particle size than those produced with HP-functional CDs. FTIR, TGA and solid-state ^13^C NMR demonstrated the adsorption of CDs on the particle surfaces. The proposed concept allows for the synthesis of silica nanoparticles with control over particle shape and size by adjusting the concentration of additives in a simple, one-pot reaction system for a wide range of applications.

## Introduction

With the availability of several types of surfactants and the increased understanding of sol–gel chemistry, numerous mesoporous silica nanoparticles (MSNs) with tunable structural properties have been developed [1–11]. Yet, there is still ongoing interest in the synthesis of tailored MSNs to optimize their performance in applications, such as catalysis, sensing, drug delivery and adsorption. So far, this has been done by tuning multiple parameters (e.g., type and concentration of surfactants and solvents used) in the synthesis of MSNs to control the shape, size and pore structure of the particles. However, controlling these particle characteristics through a single parameter has remained as a highly challenging task to date.

MSNs are commonly synthesized by surfactant-templated sol–gel reactions [12–13]. In this regard, mesoporous silica nanospheres are the most commonly used form of silica particles that are produced using a pore templating agent, such as cetyltrimethylammonium bromide (CTAB). Rod-like MSNs were synthesized by using Pluronic 123, a copolymer of ethylene and propylene oxides as porogen additive in the presence of NH_4_F and heptane [14]. The length and width of such particles could be tuned by addition of HCl. Likewise, Oden and co-workers reported a morphological change of MSNs from rods to platelets with depending on the concentration of heptane and NH_4_F [15]. MSNs were also synthesized in ellipsoidal and tubular forms using room-temperature ionic liquids (RTILs) as pore-templates [16]. Recently, single-walled carbon nanotube functionalized MSNs with grape-like structures were produced using a combination of CTAB surfactant, ethylene glycol solvent and NH_4_OH as the catalyst. MSNs were also reported in branched forms using organosilane precursors in a one-pot, CTAB-directed sol–gel synthesis [17]. For such a system, increasing the ethyl acetate concentration led to branched MSNs with ordered, 2D hexagonal pore structure growing from specific facets of the cubic MSN cores. Further, the pore structure of such particles can be engineered through various synthesis routes. Very recently, Shimogaki et al. reported the synthesis of microporous silica particles via gradual injection of tetramethoxysilane (TMOS) and the template molecule *n*-dodecylamine into the reaction system [18]. Likewise, stearylamine, which has one less alkyl group relative to CTAB, was used as the surfactant to generate microporous silica particles [19]. On the other hand, without the requirement of a surfactant, porous silica particles could also be generated through supramolecular aggregates of nonpolar cyclodextrins (CDs), which acted as porogens to create porous particles [20].

**Figure 1:**
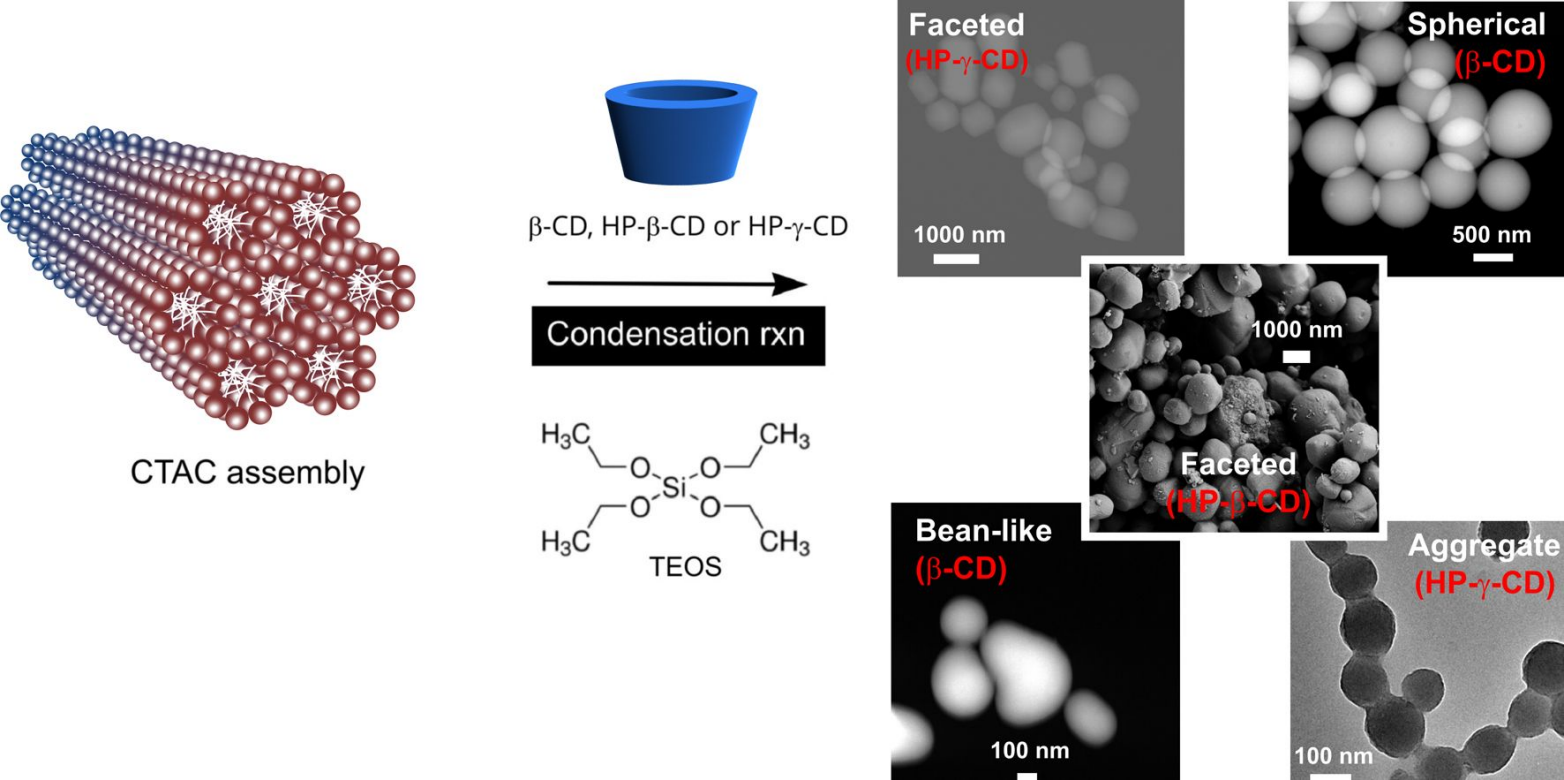
Synthesis scheme, electron microscopy images (S-TEM, TEM and SEM) of MSNs of various shapes that were synthesized in the presence of different CD types (β-CD, HP-β-CD and HP-γ-CD).

CDs are cyclic oligosaccharides with a tendency of forming aggregates in water by self-assembly. The addition of such molecules prior to silica condensation led to pores in the silica particles without the requirement of any surfactant [20]. CD assemblies lead to microporosity in the particles so that only very small molecules can pass through the pores. This inevitably induces selectivity for the molecules to be transported. In our previous paper, we observed some changes in the morphology of MSNs with CD incorporation, and used these CD-functional particles for water treatment [21]. Significant changes were observed in terms of particle size, where larger MSNs were observed in the presence of β-CD molecules. In the present paper, we performed a detailed study on the influence of CD molecules on the formation of MSNs in the presence of cetyltrimethylammonium chloride (CTAC) as the surfactant. The particles were produced by the surfactant-templated, NaOH-catalyzed silica condensation in the presence of various CD types (i.e., β*-*CD, HP-γ*-*CD and HP-β-CD). By varying the concentrations of additives and CD-type, MSNs in various shapes, such as spherical, aggregate, bean-like and faceted were generated (Figure 1). The particles were characterized in terms of morphology by SEM, TEM and STEM, the surface area by BET, chemical analysis by FTIR and solid state ^13^C NMR, thermal properties and composition by TGA, and pore structure by WAXS.

## Results and Discussion

**Figure 2:**
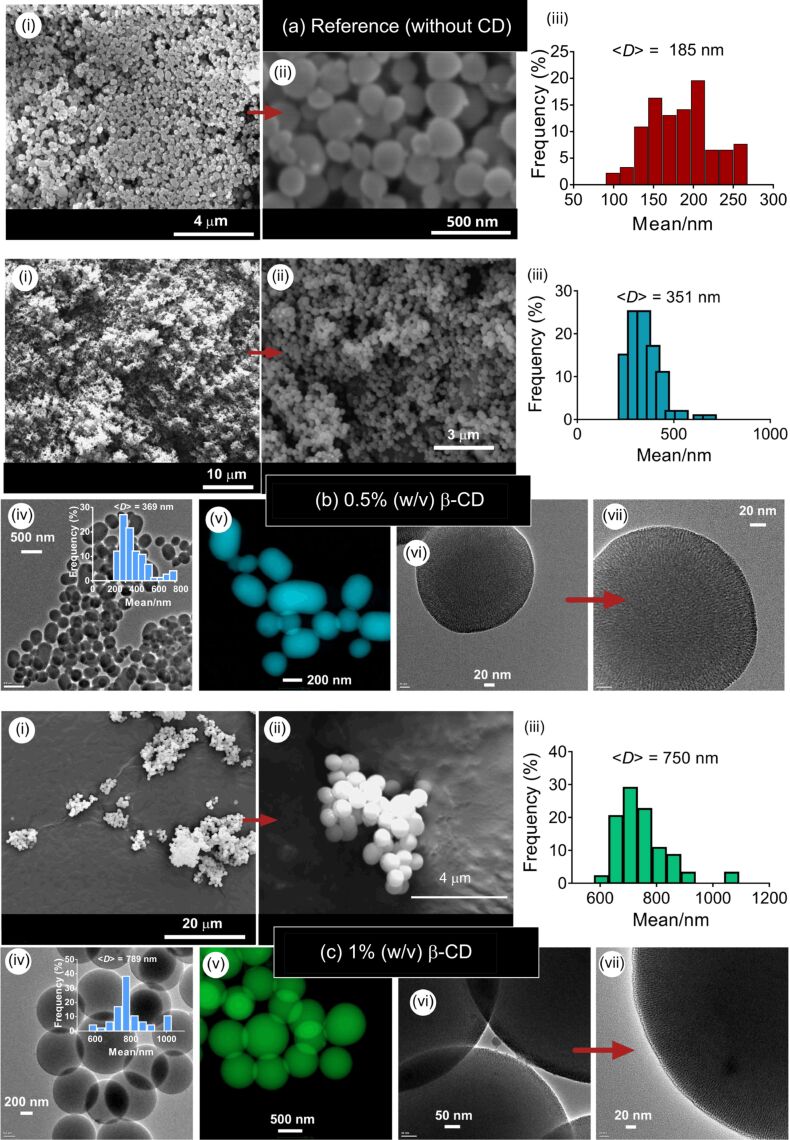
SEM (i, ii), TEM (iv, vi, vii) and colored STEM (v) images and the particle size-distribution plots (iii) of MSNs produced at two different concentrations of β-CD (MSN-1 (b) and MSN-2 (c)). (a) Reference sample (MSN-20) synthesized in the absence of CD molecules at identical concentrations of TEOS (1% (v/v)) and CTAC (0.2% (w/v)). (b) *c*_β-CD_ = 0.5% (w/v) and *r*_(CD/CTAC)_ = 5, (c) *c*_β-CD_ = 1% (w/v) and *r*_(CD/CTAC)_ = 10. Insets show the size-distribution plots of the respective particles.

Figure 2 shows the silica particles produced at β-CD concentrations of 0.5 and 1% (w/v) and constant tetraethoxysilane (TEOS) concentration of 1% (v/v). At these concentrations, single particles were synthesized. Electron microscopy analysis revealed the mixture of spherical and ellipsoid particles at 0.5% (w/v) β-CD, while only spheres were observed at a β-CD concentration of 1% (w/v). A similar spherical shape was also observed for the MSN particles synthesized by using β-CD moieties [21]. For both cases, no particle aggregation was observed. The average particle sizes (<*D*>) were, respectively, 351 nm for 0.5% (w/v) of β-CD, and 750 nm for 1% (w/v) of β-CD. The MSNs synthesized in the absence of CD moieties revealed the mixture of spherical and ellipsoid particles with a mean size of 185 nm, suggesting that the addition of β-CD leads to the formation of larger particles (Figure 2a). HRTEM images of the respective particles revealed a mesoporous structure in the particles (Figure 2b,c (vii)). Since the particles do not display any aggregation, the CTAC content was sufficiently high to generate stable particles without any aggregation.

**Figure 3:**
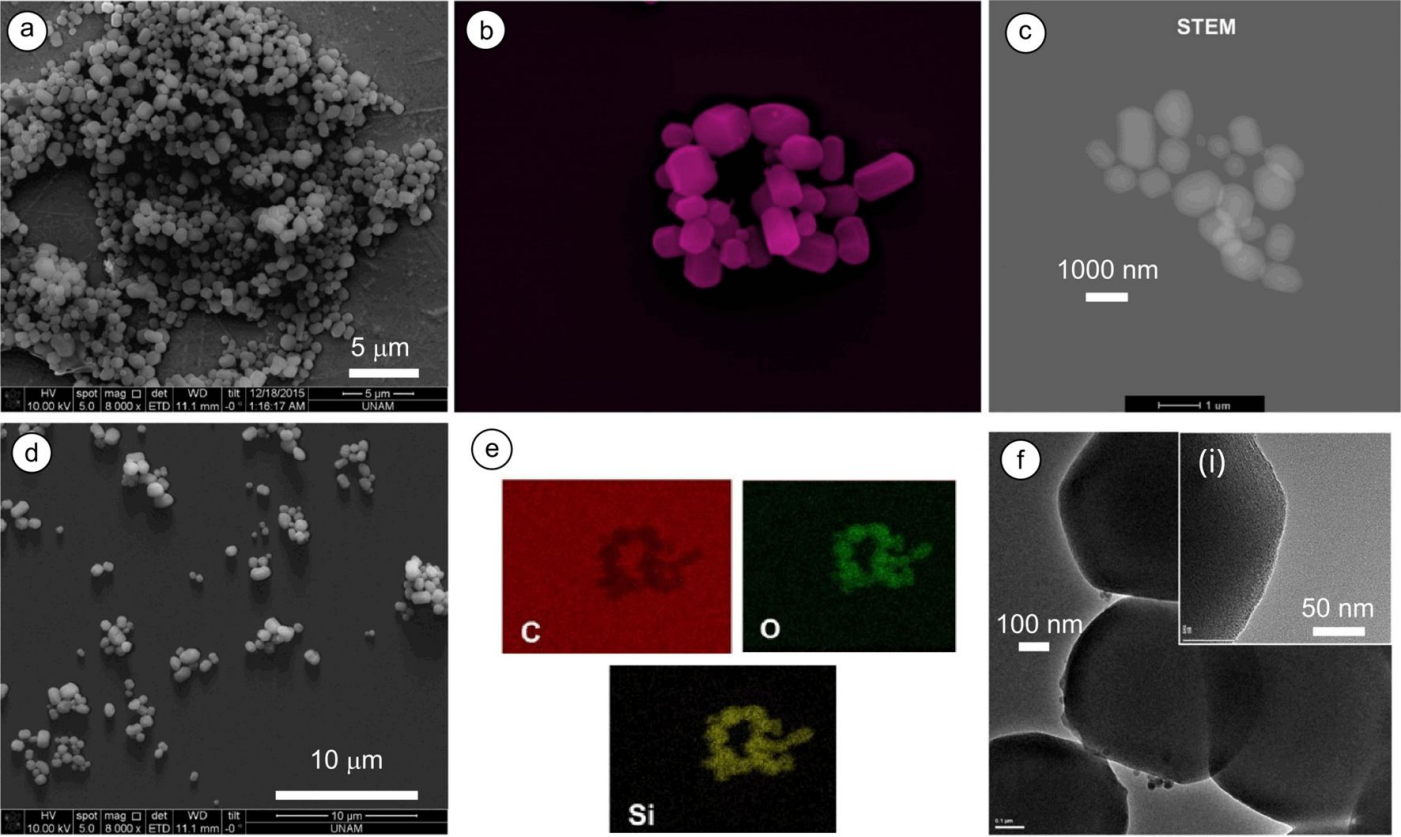
Electron microscopy images of MSNs (MSN-3) produced at 0.25% (w/v) of HP-γ-CD. (a, d) SEM photos, (b, c) STEM images, (e) EDX mappings for C, O and Si elements, and (f) TEM image of MSNs. (i) Inset shows a HRTEM image of the particle. *c*_CD_ = 0.25% (w/v) and *r*_(CD/CTAC)_ *=* 1.25.

In the following step, hydroxypropyl-functionalized CDs (i.e., HP-γ-CDs) were used in the preparation of silica particles. Unlike β-CDs, HP-CDs are amorphous, and do not show any crystalline phases owing to functionalization-induced changes in the CD structure. The solubility of CD molecules greatly varies depending on the CD structure. Pristine CDs are poorly soluble molecules due to the presence of strong intermolecular hydrogen bonding in the crystal state (e.g., the solubility of β-CD in water is 18.5 g/L) [22], while HP-functionalized CDs are highly water-soluble (according to the manufacturer “Wacker Chemie AG”, the solubility of HP-β-CD and HP-γ-CD is, respectively, 2300 g/L and 800 g/L). In water, pristine CDs form aggregates in the size range of 200–300 nm depending on the number of glucose units in the structure [23–24]. The aggregation is mainly governed by hydrogen bonds between the hydroxyl moieties. After the functionalization with HP motifs, no significant aggregation was observed for partially substituted HP-β-CDs at low concentration (12 mM) [25]. It is thus expected that the HP-γ-CD incorporation prior to the condensation reactions should lead to substantial changes in shape and pore structure of the particles relative to the particles synthesized with pristine CDs. Figure 3 shows MSNs synthesized using HP-γ-CDs of 0.25% (w/v)). Intriguingly, faceted MSNs were formed with a mean size of 825 nm. The variations in CD-type and concentration led to marked changes in particle morphology, thus allowing for the synthesis of nanoparticles in a variety of shapes (e.g., from round to ellipsoid and even faceted). This shape change could be attributed to the self-assembly of CDs after modification with HP groups. These molecules do not aggregate like β-CD, which might affect the particle formation, and eventually lead to the particles in different shapes and pore structures. For detailed analysis of the faceted particles, TEM, STEM and EDX analyses were performed. Figure 3f shows the TEM images of the nanoparticles with microporous structure. All particles had irregular shapes with many smooth faces. The surface area and pore volume of these particles were 764.38 m^2^/g and 0.853 cm^3^/g, respectively, while the average pore radius was 3.91 Å (Figure S1c,d, Supporting Information File 1). These particles with smooth faces and microporous structure are highly interesting forms of silica nanoparticles. Particles with a similar structure synthesized using NH_4_OH-catalyzed silica condensation at low surfactant contents by two-step reactions –(i) nuclei formation and (ii) particle growth– in a dilute alkaline solution were previously reported [26]. For the generation of different shapes and faceted morphology of mesoporous silica, Ozin and co-workers reported that the shape of mesoporous silica largely depends on the degree of curvature and the accretion-type induced by various topological defects in the SiO_2_–CTAC precursor particles that direct the growth of hexagonal mesoporous silica toward specific morphology [27–28]. Suzuki et al. also reported the synthesis of faceted MSNs using TEOS, CTAB and a copolymer of ethylene and propylene oxide (Pluronic F127), where the copolymer acted as a second surfactant and suppressed the grain growth and stabilized particle structure [29]. This scenario can also be credited to the formation of faceted MSNs in the presence of HP-CDs, where CDs involved as the second surfactant during particle formation, and directed the growth of silica particles in a particular shape. EDX analysis of particles demonstrated the presence of carbon in addition to oxygen and silicone, suggesting physical attachment of CDs. FTIR was used to confirm adsorbed CDs on silica particles (Figure S1b, Supporting Information File 1). The peaks at 2860 and 2930 cm^−1^ are associated with the C–H stretching of CTAC molecules, while the peak at 460 cm^−1^ is assigned to the asymmetric vibration of Si–O–Si. The Si–OH bond vibration can be seen at 802 cm^−1^. The presence of CD moieties is confirmed by the peaks at 1030, 1080 and 1155 cm^−1^ for the stretching vibration of C–C and C–O bonds and the asymmetric stretching of C–O–C [30]. But, these peaks are overlapped by a broad stretching vibration peak of Si–O–Si at 1100 cm^−1^. Comparable FTIR spectra were observed for the CD-functionalized silica particles, suggesting a physical adsorption of CDs at the particle surface [21]. Thermogravimetric analysis (TGA) of the particles revealed a small mass loss (ca. 2% (w/v)) above 300 °C due to adsorbed CD moieties (Figure S2, Supporting Information File 1).

**Figure 4:**
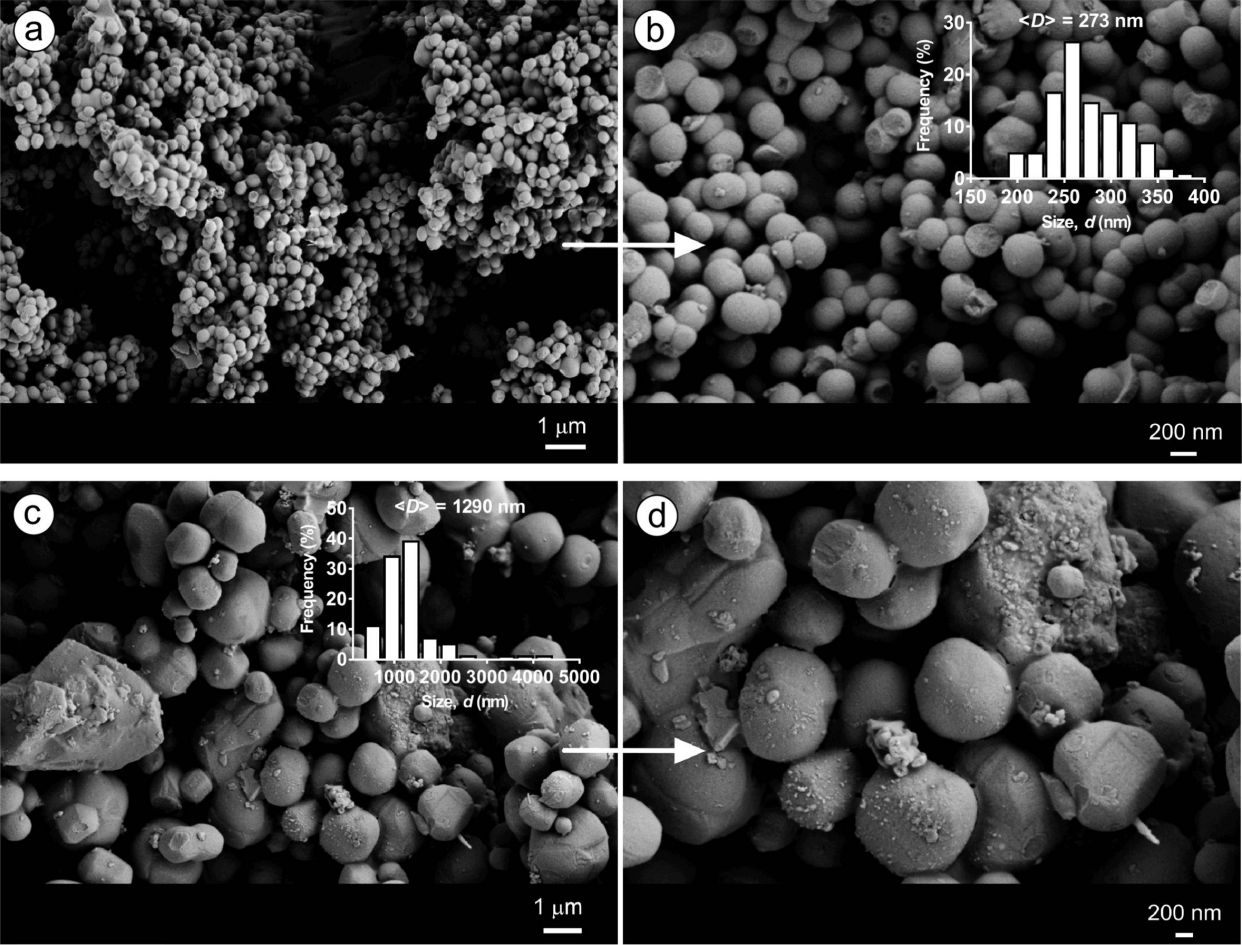
SEM images of MSNs produced using β-CD (MSN-4 (a, b)) and HP-β-CD (MSN-5 (c, d)). (a, b) *c*_β-CD_ = 0.25 (w/v) and *r*_(CD/CTAC)_ = 2.5, and (c, d) *c*_HP-β–CD_ = 0.25% (w/v) and *r*_(CD/CTAC)_ = 2.5. Insets show the size-distribution plots of the respective particles.

For further confirmation of shape transformation of the particles from round to multifaceted ones with HP-functional CDs, MSNs were also synthesized using β*-*CD and HP-β-CD. Figure 4 shows the particles produced at 0.25% (w/v) of CDs and CTAC concentration of 0.10% (w/v). The particles synthesized with β-CD yielded spherical nanoparticles with a size of ca. 270 nm, whereas multifaceted microparticles with a mean particle size of 1290 nm were observed with HP-β-CD. This is line with the findings of particles produced using HP-γ-CD at high surfactant contents indicating that the multifaceted morphology with HP-functionalized CDs is common (Figures 2–4). Also, the use of HP-functionalized CDs clearly leads to bigger particles than the particles synthesized with β-CD at identical concentrations (see Table S1, Supporting Information File 1).

**Figure 5:**
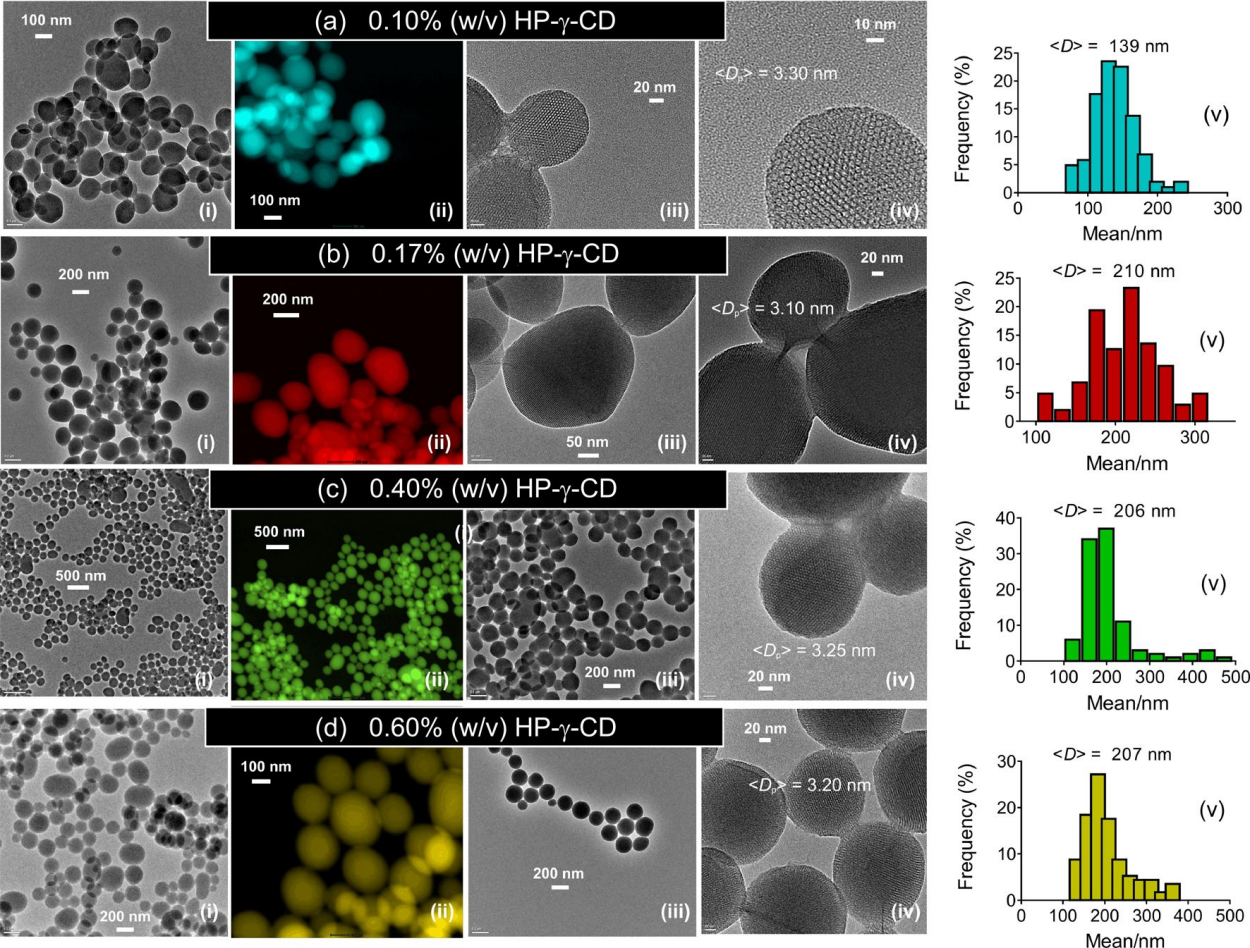
TEM (i, iii, iv) and colored STEM (ii) images, and as well as the particle size-distribution plots (v) of MSN particles (MSN-6-9) produced at various concentrations of HP-γ-CD (a–d). *c*_CD_ = 0.10–0.60% (w/v), *c*_CTAC_ = 0.20% (w/v) and *r*_(CD/CTAC)_ = 0.50 (a), 0.85 (b), 2 (c) and 3 (d). <*D*_p_> denotes the mean pore size calculated from the TEM images.

The influence of the HP-γ-CD concentration on the particle morphology was explored by increasing the CD content from 0.10 to 0.60% (w/v) at a CTAC concentration of 0.20% (w/v) and a TEOS concentration of 1% (v/v). Figure 5 shows the TEM and STEM images of the particles. Under all conditions, MSNs were obtained where the particle shape transformed from single particles to aggregates depending on the CD content used. At low HP-γ-CD concentrations, mixed particles with spherical and faceted shape were formed. However, the particle morphology changed to spherical with an increase of the CD content. At higher CD concentrations, the formation of single particles was apparent. This might be attributed to high water solubility of HP-γ-CDs. These moieties might shield the particle surface from fusion with other particles. Interestingly, at low CD concentrations, the resultant particles exhibited smooth faces. The mean particle size increased from 139 to 207 nm with the HP-γ-CD content increasing from 0.1 to 0.6% (w/v), while the mean pore size of the respective particles remained nearly stable at 3.20 ± 0.10 nm. Similarly aggregated MSNs were observed for the particles synthesized with HP-β-CDs [21], suggesting that both HP-functionalized CDs (either β- or γ-CDs) have a similar influence on the particle formation.

**Figure 6:**
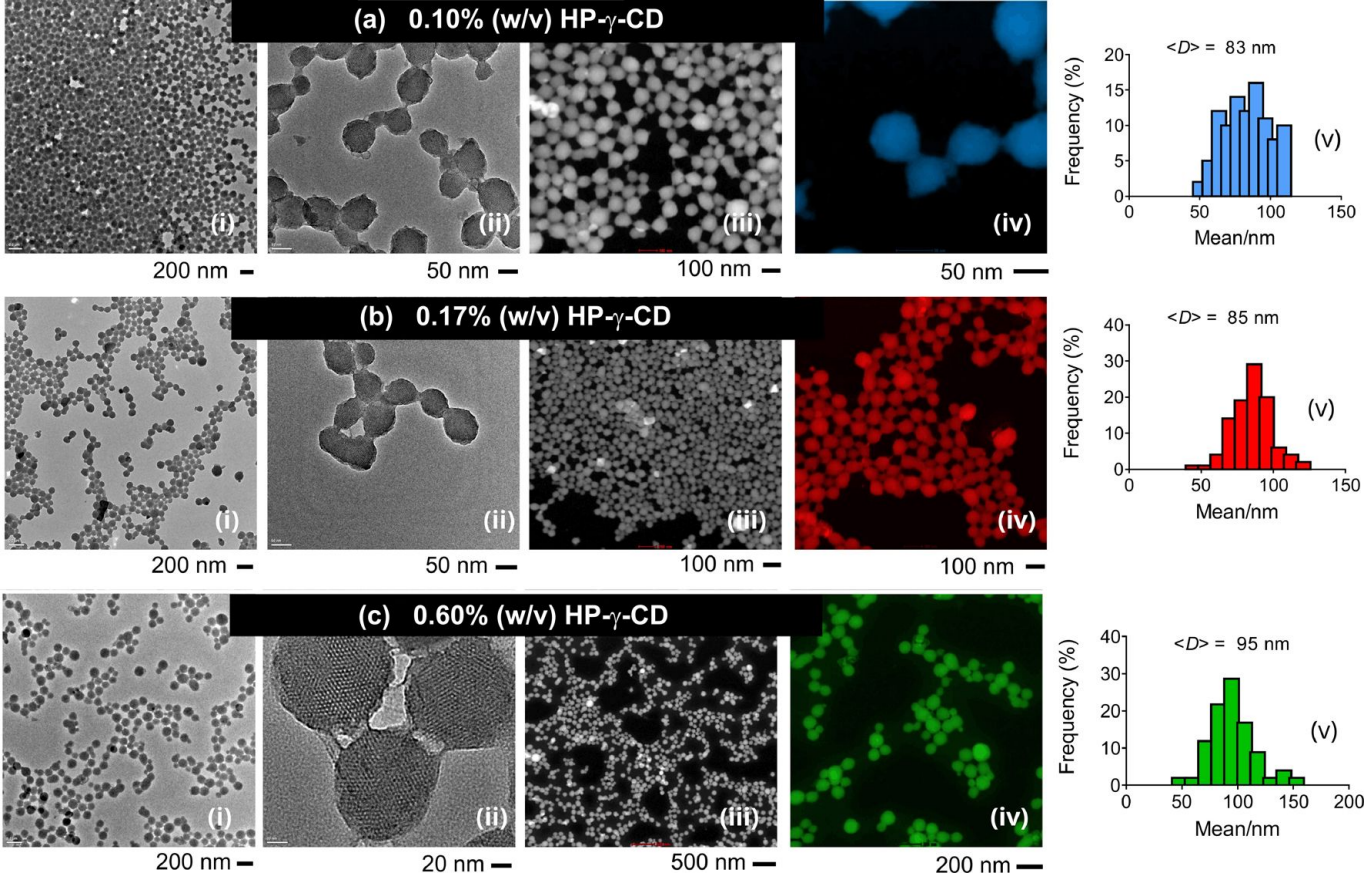
TEM (i, ii), colored STEM (iii, iv) images, and the particle size-distribution plots (v) of MSNs (MSN-10-12) produced at various HP-γ-CD concentrations. *c*_CTAC_ = 0.40% (w/v), *c*_HP-γ-CD_ = 0.1 (a), 0.17 (b) and 0.6% (w/v) (c), and *r*_(CD/CTAC)_ = 0.25 (a), 0.42 (b) and 1.5 (c). The average particle size was calculated from the diameters of the single particles.

Similar experiments were conducted by increasing the HP-γ-CD concentration (0.1–0.6% (w/v)) at identical CTAC content (0.4% (w/v), Figure 6). The particle size clearly decreased as the CTAC content increases. This agrees with previous results that suggested an influence of CTAC on the particle size; increasing CTAC concentration led to smaller particles. Under all conditions, MSN aggregates were formed with chemically fused domains of silica due to the high CTAC content. TEM analysis of the nanoparticles revealed a mesoporous structure at all compositions studied. No significant change in the particle shape was observed apart from a slight increase in the particle size from 83 to 95 nm when the HP-γ-CD content was increased from 0.10 to 0.60% (w/v).

**Figure 7:**
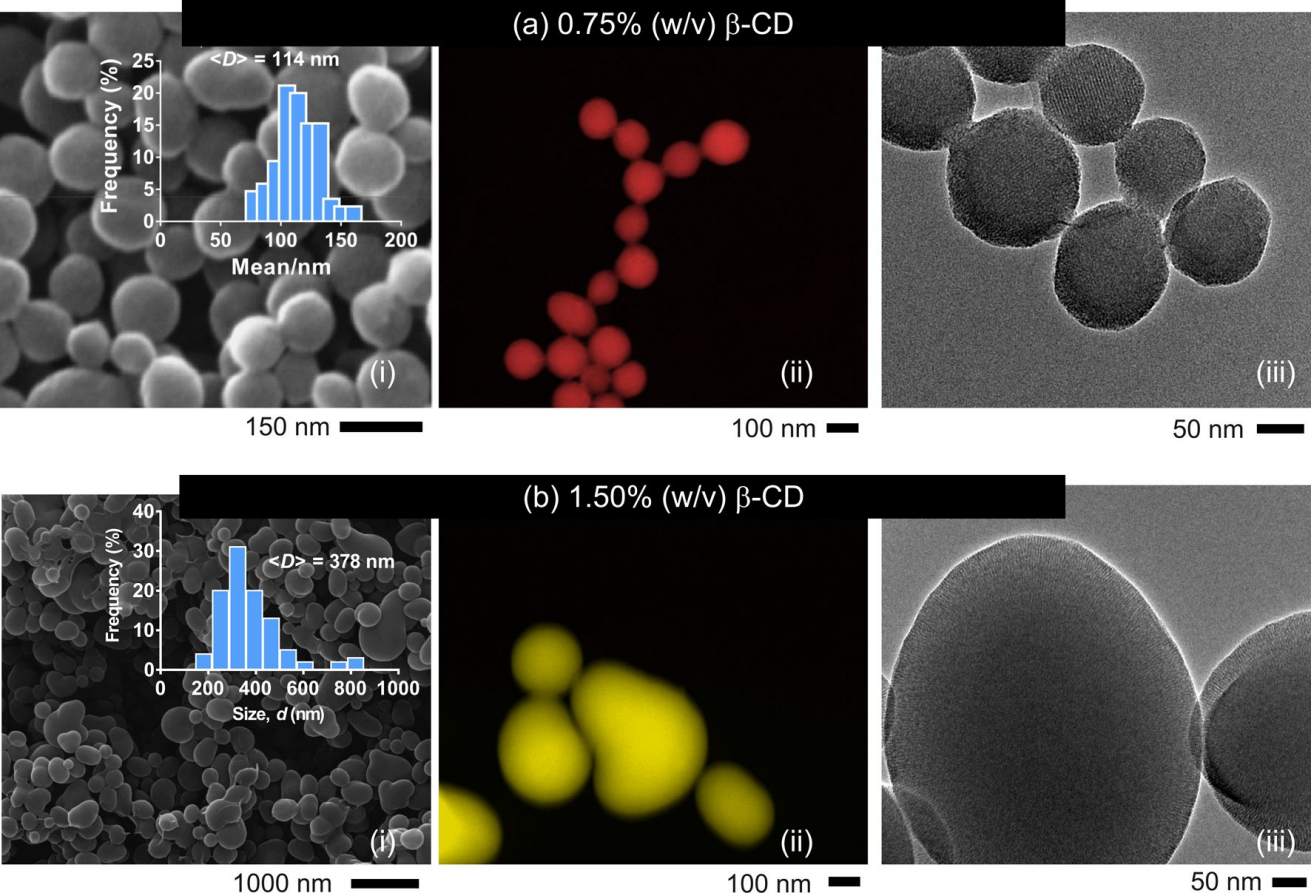
SEM (i), colored STEM (ii) and TEM (iii) images of MSNs produced at various β-CD contents; (a) MSN-14 (*c*_β-CD_ = 0.75% (w/v), *r*_(CD/CTAC)_ = 3.75), and (b) MSN-15 (*c*_β-CD_ = 1.50% (w/v)), *r*_(CD/CTAC)_ = 7.5). Insets (a(i), b(i)) show the size-distribution plots of the respective particles.

MSNs were also synthesized at low CTAC content (0.2% (w/v)) and two different β-CD concentrations (0.75 and 1.5% (w/v)). Figure 7 displays the TEM and STEM images of the respective particles, where inter-linked silica nanospheres were generated at a β-CD content of 0.75% (w/v). In contrast, single MSNs with bean-like structure were observed with a β-CD content of 1.5% (w/v). This agrees with Figure 5, where aggregate particles were observed at low CD concentrations. The mean particle size increased from 114 to 345 nm (Figure 7 and Figure S3, Supporting Information File 1). This dramatic increase in particle size with increasing β-CD content is in line with our previous report, which revealed the formation of larger particles when β-CD molecules were used [21]. The particle size decreased to 112 nm when the β-CD concentration was reduced to 0.25% (w/v). Like the particles synthesized at 0.75% (w/v) β-CD, MSNs prepared at 0.25% (w/v) revealed particle aggregates (Figure S4, Supporting Information File 1). HRTEM images of the particles evidenced porosity in both particles.

**Figure 8:**
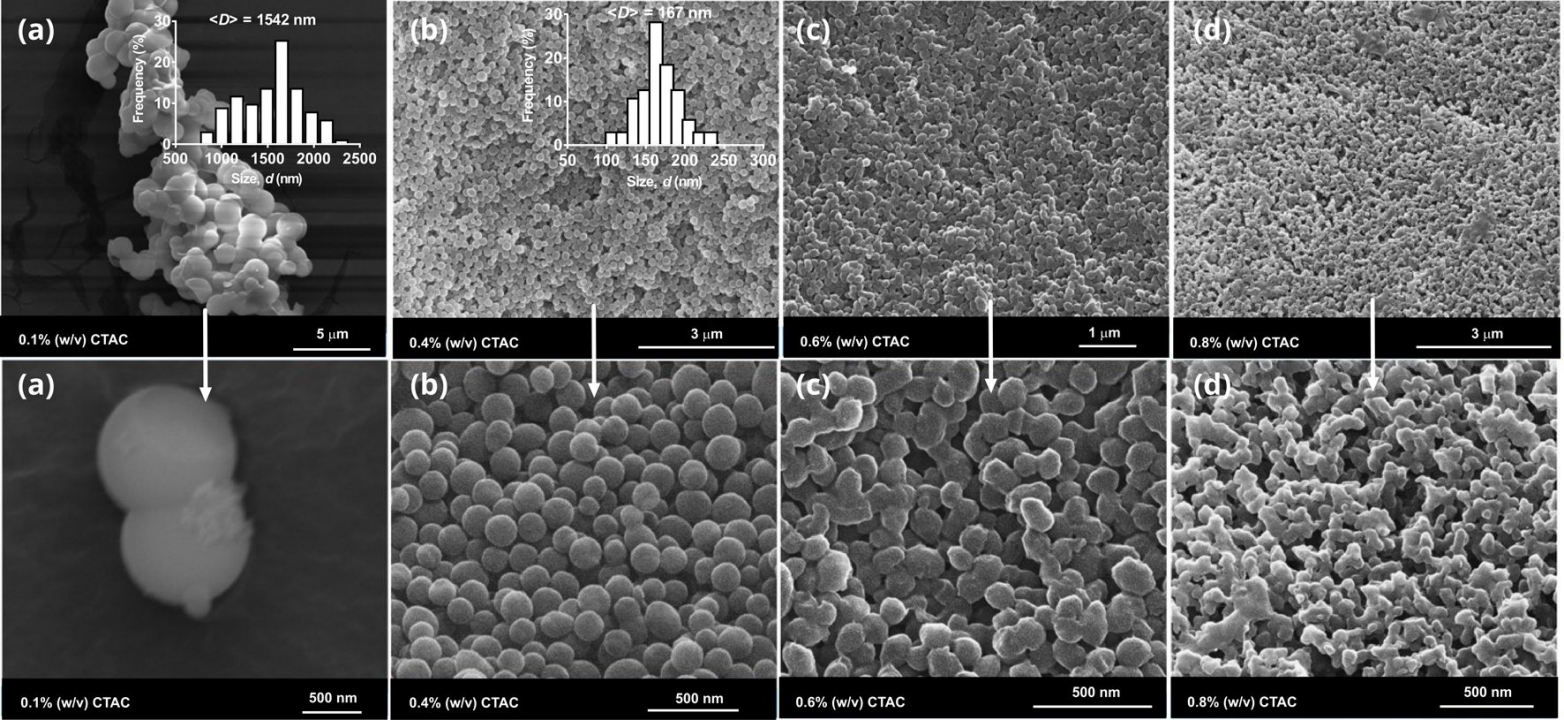
SEM images of the nanoparticles produced at various CTAC concentrations: (a) MSN-16 (*r*_(CD/CTAC)_ = 10), (b) MSN-17 (*r*_(CD/CTAC)_ = 2.5), (c) MSN-18 (*r*_(CD/CTAC)_ = 1.66) and (d) MSN-19 (*r*_(CD/CTAC)_ = 1.25); *c*_β-CD_ = 1% (w/v). The size distribution plots of the respective particles were shown as insets.

The nanoparticles were also synthesized at various CTAC concentrations at the constant β-CD concentration of 1% (w/v). Figure 8 shows the SEM images of the respective particles. At low CTAC concentration (0.10% (w/v)), the formation of large connected particles was observed with a mean particle size of 1500 nm. With an increased CTAC concentration of 0.40% (w/v), silica nanospheres with a size of about 170 nm were produced. A further increase in the CTAC concentration led to smaller, connected nanoparticles in the range of 110–130 nm. A similar change in particle morphology was reported for the CD-free MSN samples synthesized at various CTAB contents, where the sample texture showed a dramatic change with the CTAB content used [31].

**Figure 9:**
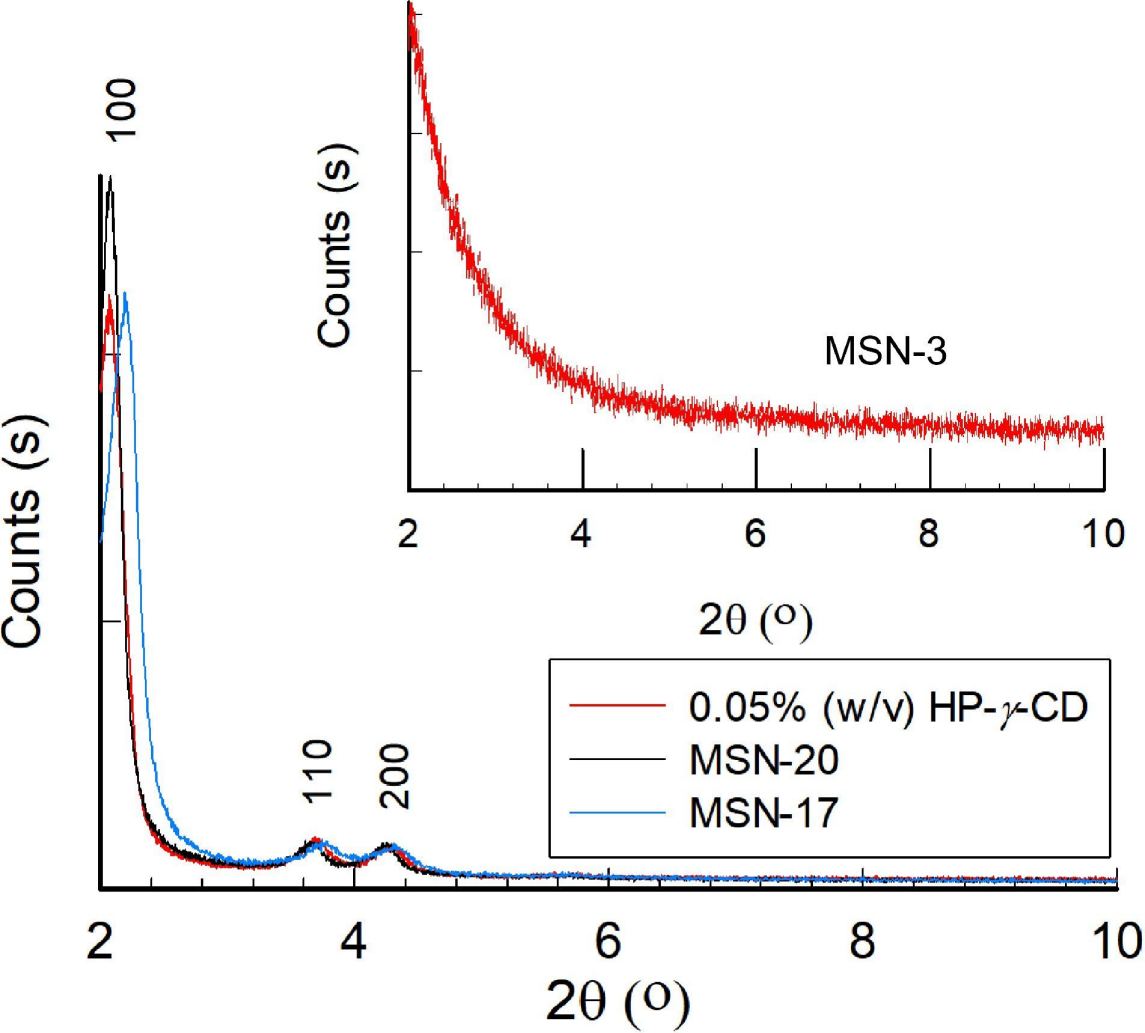
Wide-angle XRD patterns of the MSNs in the 2θ range of 2–10°. MSN samples synthesized with HP-γ-CD (0.05% (w/v)) and β-CD (1% (w/v)) (MSN-17). Inset shows the XRD pattern of MSN-3.

The pore structure of some MSNs was explored by WAXS in which sharp peaks related to periodic mesoporosity were observed at 2θ angles between 2 and 10° (Figure 9), which agrees with the TEM results. XRD peaks for the MSN synthesized in the absence of CDs (MSN-Blank) and the MSN samples prepared in the presence of 0.05% (w/v) HP-γ-CD and 1% (w/v) β-CD appeared at 2.29° (100), 3.77° (110), and 4.35° (200). These are typical peaks of periodical mesopores in MSNs [32]. The XRD pattern of the multifaceted sample did not reveal any peak related to the mesoporosity in the 2θ range of 2–10°. This might be attributed to the presence of micropores smaller than 1 nm as confirmed by BET (*D* = 7.82 Å). The particles synthesized at low HP-β-CD content revealed a mesoporous structure. Thus, the pore structure of MSNs clearly displays significant changes with the CD-type used. β-CD moieties are highly crystalline compounds and form ordered multimolecular structures, while HP-γ-CD has amorphous characteristics with different aggregation behavior (Figure S5, Supporting Information File 1). Therefore, the shifts in the WAXS patterns could be attributed to the alterations in the pore architecture, which was directed by the self-assembly of β-CD during the particle formation.

The pore size and volume of the particles were measured using the Brunauer–Emmett–Teller (BET) method. Figure S6 (Supporting Information File 1) shows the nitrogen adsorption–desorption isotherms and the pore size diagrams of MSNs. The pristine MSN sample, which does not have any CD moieties, has a mean pore size of 2.64 nm and a surface area of 1374.25 m^2^/g. BET analysis of the multifaceted silica particles synthesized with 0.25% (w/v) HP-γ-CD revealed a microporous structure with a mean pore size of 0.78 nm while the surface area of the respective particles was 764.38 m^2^/g. This is in line with the HRTEM analysis of the respective particles (Figure 3f). Even though the pore size is significantly smaller, the pore volume was increased from 0.791 to 0.853 cm^3^/g. The presence of the CD moieties on the particles was confirmed by thermogravimetric analysis (TGA). TGA curves of MSNs clearly demonstrated mass loss at ca. 320 °C for the CD-functionalized MSNs because of adsorbed CDs (Figure S7, Supporting Information File 1). In contrast, MSN particles produced without using CD moieties did not show any weight loss in the same temperature range as silicone oxide (SiO_2_) is a thermally stable material. The presence of adsorbed CD moieties on the particles was confirmed by solid-state ^13^C NMR analyses. Figure S8 (Supporting Information File 1) shows the CP/MAS ^13^C NMR spectra of the MSNs prepared with β-CD and HP-γ-CD moieties. The β-CD-functionalized MSN sample shows typical peaks of the respective carbon peaks of β-CD; C-1 (97–106 ppm), and C-2–5 (78–86 ppm), while the HP-γ-CD-functionalized sample displays dominant C peaks at 19.7, 30, 54 and 68 ppm, which could be ascribed to the carbon atoms of the HP-CDs. These silica particles with adsorbed CD motifs can be used for desired applications as previously implemented for water-treatment [21].

## Conclusion

This paper demonstrates the effect of CD addition on the formation of silica nanoparticles. In our previous paper, we reported the synthesis of CD-functionalized MSNs and used them for the removal of polycyclic aromatic hydrocarbons from water [21]. In this paper, we investigated the influence of CD molecules on the formation of MSNs at various concentrations of the precursors. The nanoparticles were synthesized by the CTAC-templated, NaOH-catalyzed silica condensation in the presence of CD moieties (pristine or HP-functionalized ones). By varying the formulation parameters (CD content and type), nanoparticles were produced in various shapes (aggregates, spherical, bean-like and faceted). TGA, ^13^C MAS NMR and FTIR analyses confirmed a physical adsorption of CD molecules. The surface area of MSNs varied depending on the formulation parameters. The particle shape was influenced by the concentrations of both CD and CTAC and as well as the ratio between them. The variation of the CD type during the particle synthesis led to intriguing changes in particle morphology. In general, faceted particles were obtained with HP-CDs, while spherical ones were obtained with β-CD. The particles became smaller with increasing CTAC content. A further increase of CTAC content led to particle aggregation. On the other hand, increasing the CD concentration led to larger particles.

## Experimental

### Materials

Ethanol (EtOH, >99%) and tetraethyl orthosilicate (TEOS, 98%) were purchased from Sigma-Aldrich. Hexadecyltrimethylammonium chloride (CTAC, >98%) was received from TCI Chemicals (Germany). β-Cyclodextrin (β-CD, (Cavamax^®^ W7)), hydroxypropyl-β-cyclodextrin (HP-β-CD (Cavasol^®^ W7 HP), molar substitution per anhydroglucose unit: 0.60) and hydroxypropyl-γ-cyclodextrin (HP-γ-CD (Cavasol^®^ W8 HP), molar substitution per anhydroglucose unit: 0.58–0.73) were kindly donated by Wacker Chemie AG (Germany). Sodium hydroxide (NaOH) was purchased from Merck.

### Synthesis of silica nanoparticles

The synthesis of silica nanoparticles was carried out following our previous method [21]. Briefly, an aqueous solution of CTAC (in 97 mL water), NaOH (0.7 mL, 2 N), CD (at various concentrations) and TEOS (1 mL) was continuously stirred at 80 °C for 3 h. For the synthesis of pristine MSNs, the particles were synthesized without CD addition. The nanoparticles were later purified by washing with a mixed solution of acetic acid and methanol with a ratio of 1:3 to remove weakly adsorbed CDs and CTAC moieties from the surface and pores of particles. Table S1 (Supporting Information File 1) gives the composition and characteristics of MSNs.

### Characterization

The particles were imaged by scanning electron microscopy (SEM, Quanta 200 FEG, FEI). The average particle diameters (<*D*>) and their distributions were calculated by analysing ca. 100 particles from SEM images using ImageJ software (NIH, Bethesda, MD, USA). Energy-dispersive X-ray spectroscopy (EDX) was used to determine the elemental composition of the MSN samples at 30 kV and 4.5 mA. Transmission electron microscopy (TEM, FEI Tecnai G2F30) analysis was performed on the particles, which were dispersed in water, and a tiny droplet was dried on a TEM grid. The particles were imaged at 300 kV. STEM images were captured using a high-angle annular dark field (HAADF) detector. The chemical compositions of nanoparticles were explored by solid-state ^13^C NMR using an Inova 500 MHz NMR Varian system fitted with a Jacobsen brand CP/MAS probe. Two thousand scans were acquired for measurements. Fourier transform infrared (FTIR) spectra of MSNs were recorded on a Bruker-VERTEX 70 spectrometer. The spectra were taken at a resolution 4 cm^−1^ after 128 scans accumulation for an acceptable signal/noise ratio. The surface area and pore volume were measured by a Quantachrome NOVA 2200e series surface analyzer. The particles were outgassed for 24 h at 110 °C. The adsorption isotherms of nitrogen at 77K were investigated through Brunauer–Emmett–Teller (BET) measurements in the *p*/*p*_0_ range of 0.05–0.3. The pore size distribution was obtained from the adsorption isotherms by the Barrett–Joyner–Halenda (BJH) method. Wide-angle X-ray scattering (WAXS) experiments were performed using a PANalytical X'Pert Pro MPD, which was powered by a Philips PW3040/60 X-ray generator fitted with an X'Celerator detector. Diffraction data was acquired by exposing samples to Cu Kα X-ray radiation. X-rays were generated from a Cu anode that was supplied with 40 kV and current of 40 mA. The data were collected over the 2θ range of 2–10° using the scanning X’Celerator detector system. All scans were carried out in continuous mode. The data were analysed by using X’Pert Highscore Plus software (version 2.0).

## Supporting Information

Supporting Information contains composition and characteristics of MSNs (Table S1), as well as additional characterization data of the MSNs by SEM, TEM, STEM, BET, FTIR, solid-state ^13^C NMR, TGA and WAXS.

File 1 Additional experimental data.
