# Supplementary material for: Cyclodextrin-assisted synthesis of tailored mesoporous silica nanoparticles
Source: Beilstein J Nanotechnol. 2018 Feb 22;9:693–703. doi: 10.3762/bjnano.9.64 (PMC5827706; doi:10.3762/bjnano.9.64)
Supplement: File 1 — Additional experimental data. [file Beilstein_J_Nanotechnol-09-693-s001.pdf]

Supporting Information

for

Cyclodextrin-assisted synthesis of tailored mesoporous silica nanoparticles

Fuat Topuz*[1,2] and Tamer Uyar*,§[1,3]

Address: [1]UNAM-National Nanotechnology Research Center, Bilkent University, 06800 Ankara, Turkey; [2]Faculty of Engineering and Natural Sciences, Sabanci University, 34956 Istanbul, Turkey and [3]Institute of Materials Science and Nanotechnology, Bilkent University, 06800 Ankara, Turkey

Email: Fuat Topuz* - fuat.topuz@rwth-aachen.de;
Tamer Uyar* - tamer@unam.bilkent.edu.tr

* Corresponding author
§ Phone: +90 (312) 290 8987

Additional experimental data

Table S1: The composition and characteristics of the MSNs.

Sample Code	CD-Type	CD (% (w/v))	TEOS (% (v/v)	$r_{CD/CTAC}$ (w/w)	NaOH 2M (mL)	Water (mL)	Synthesis Temp (^{o}C)	Particle Shape[a]	Particle (nm)[b]
MSN-1	β	0.50	1	5	0.7	96	80	Spherical - Ellipsoid	351
MSN-2	β	1	1	10	0.7	96	80	Spherical	750
MSN-3	HP-γ	0.25	1	1.25	0.7	96	80	Faceted	825
MSN-4	β	0.25	1	2.50	0.7	96	80	Spherical	273
MSN-5	HP-β	0.25	1	2.50	0.7	96	80	Faceted	1290
MSN-6	HP-γ	0.10	1	0.50	0.7	96	80	Spherical-Faceted	139
MSN-7	HP-γ	0.17	1	0.85	0.7	96	80	Spherical-Faceted	210
MSN-8	HP-γ	0.40	1	2	0.7	96	80	Spherical	206
MSN-9	HP-γ	0.60	1	3	0.7	96	80	Spherical	207
MSN-10	HP-γ	0.10	1	0.25	0.7	96	80	Spherical	83
MSN-11	HP-γ	0.17	1	0.42	0.7	96	80	Spherical	85
MSN-12	HP-γ	0.60	1	1.50	0.7	96	80	Spherical	95
MSN-13	β	0.25	1	1.25	0.7	96	80	Spherical	112

MSN-14	β	0.75	1	3.75	0.7	96	80	Spherical	125
MSN-15	β	1.5	1	7.50	0.7	96	80	Bean-like	484
MSN-16	β	1	1	10	0.7	96	80	Spherical (linked)	1542
MSN-17	β	1	1	2.50	0.7	96	80	Spherical (monodisperse)	167
MSN-18	β	1	1	1.66	0.7	96	80	Spherical (linked)	130
MSN-19	β	1	1	1.25	0.7	96	80	Spherical (linked)	110
MSN-20	—	—	1	0	0.7	96	80	Spherical-Ellipsoid	185

[a]The particle shape was evaluated through SEM or TEM analysis. [b]Particle size was determined through SEM or TEM images over single particles.

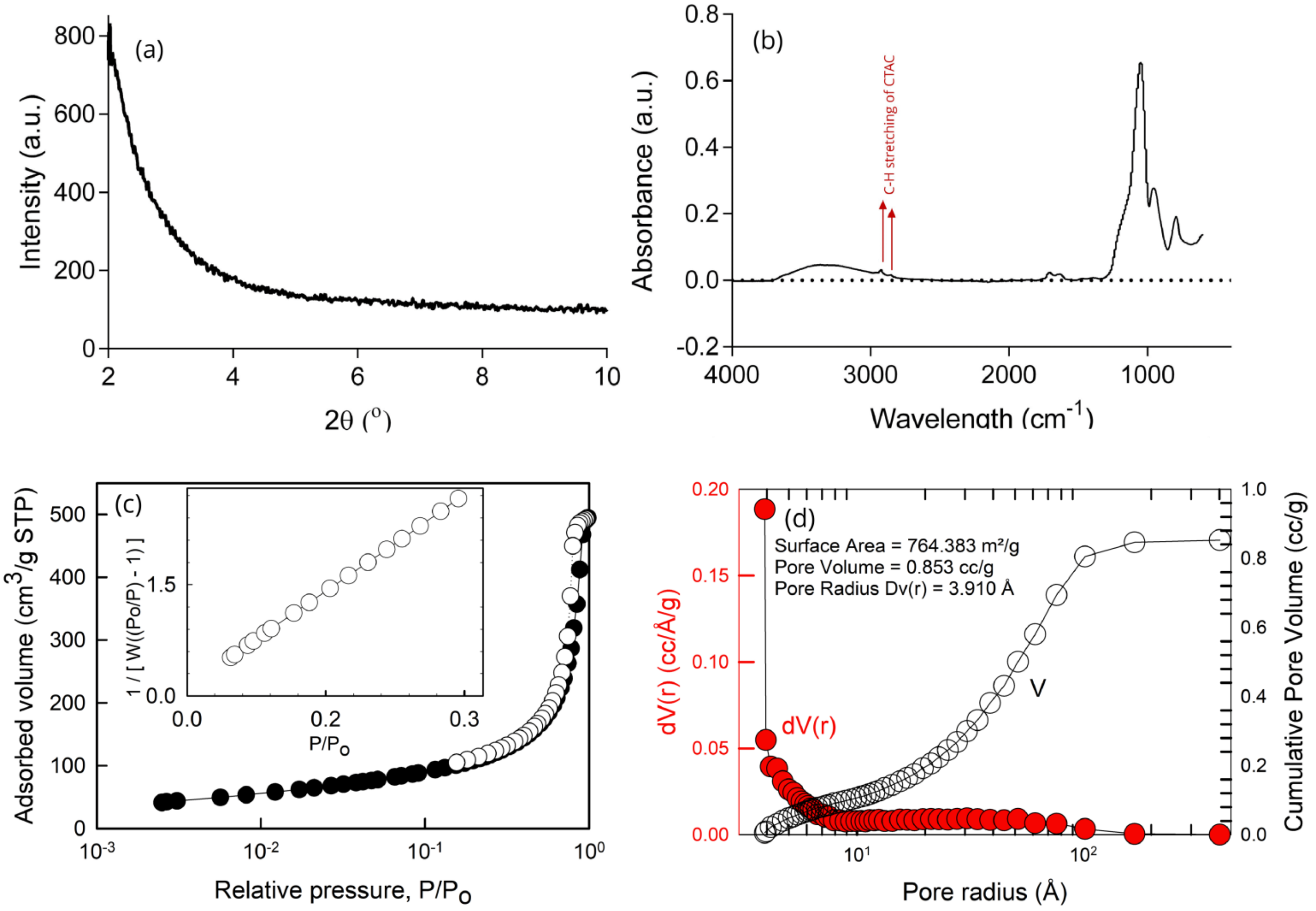

Figure S1: (a) XRD pattern and (b) FTIR spectrum of MSN-3. $c_{HP\text{-}\gamma\text{-}CD}$ = 0.25% (w/v) and $r_{CD/CTAC}$= 1.25. (c) The nitrogen adsorption–desorption isotherms and (d) BJH pore-size distrubution of MSN-3. Inset shows the multi-point BET plot.

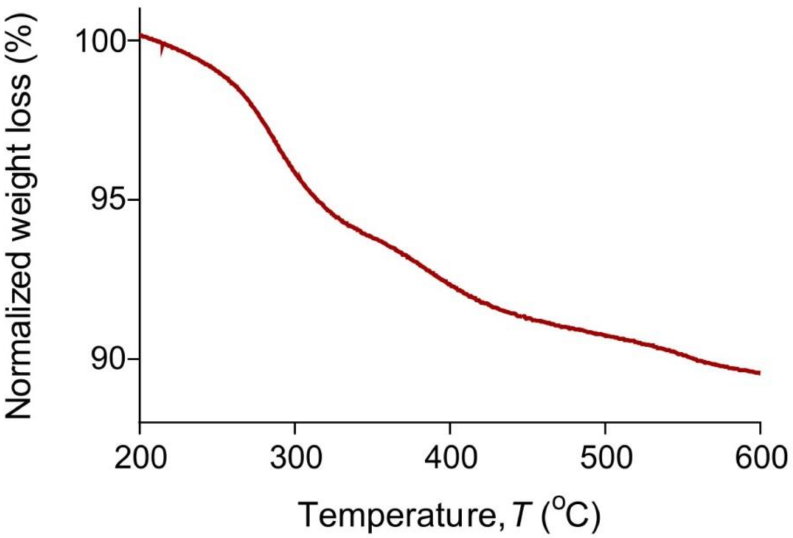

Figure S2: TGA of MSN sample produced at 0.25% (w/v) of HP-γ-CD (MSN-3). c_{CD} = 0.25% (w/v), $r_{(CD/CTAC)}$ = 1.25 and T = 80 °C.

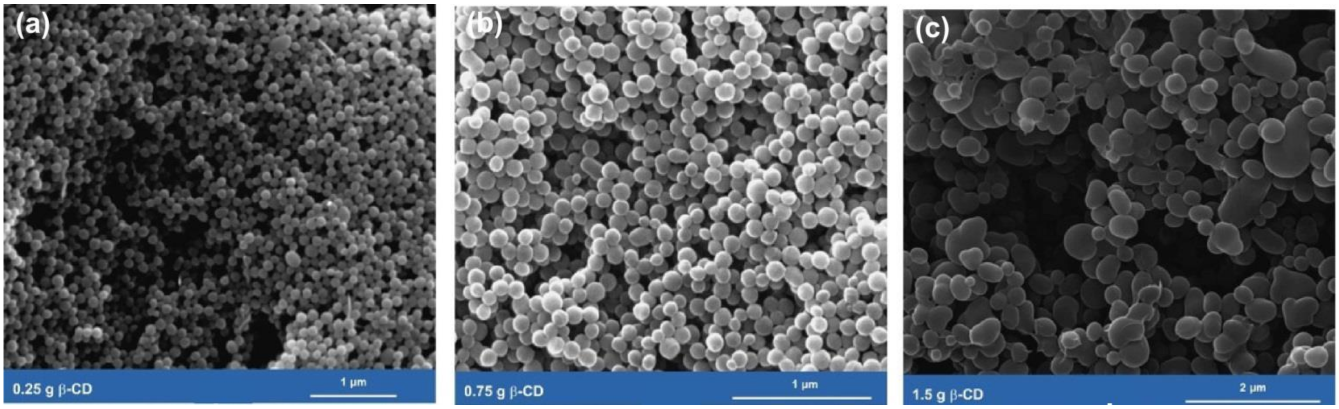

Figure S3: SEM images of the nanoparticles produced at various β-CD concentrations; MSN-13 ($c_{\beta\text{-CD}}$ = 0.25% (w/v) and $r_{CD/CTAC}$ = 1.25) (a), MSN-14 ($c_{\beta\text{-CD}}$ = 0.75% (w/v) and $r_{CD/CTAC}$ = 3.75) (b) and MSN-15 ($c_{\beta\text{-CD}}$ = 1.5% (w/v) and $r_{CD/CTAC}$ = 7.50) (c).

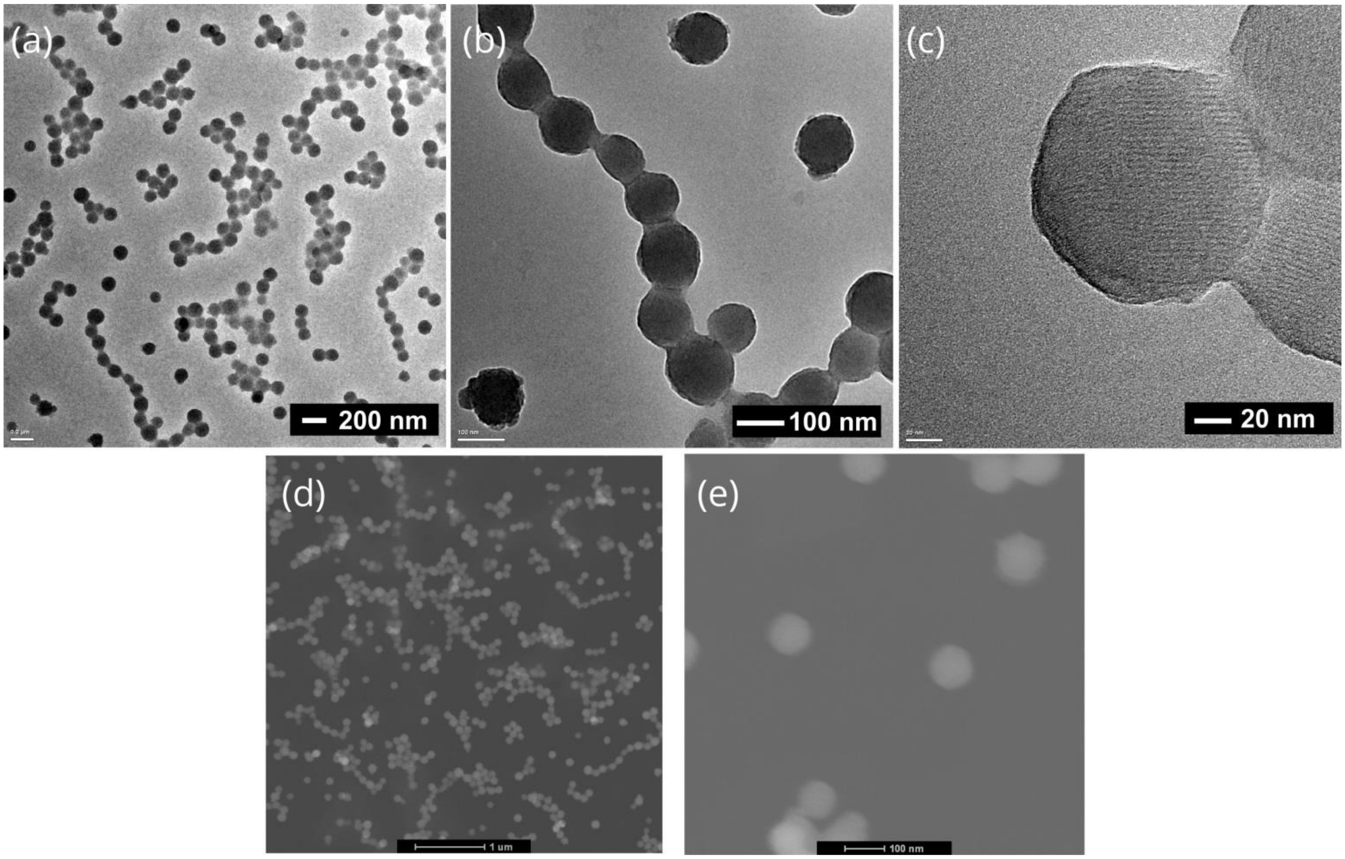

Figure S4: TEM (a–c) and STEM (d, e) images of MSNs (MSN-13). $c_{\beta\text{-CD}}$ = 0.25% (w/v) and $r_{CD/CTAC}$ = 1.25.

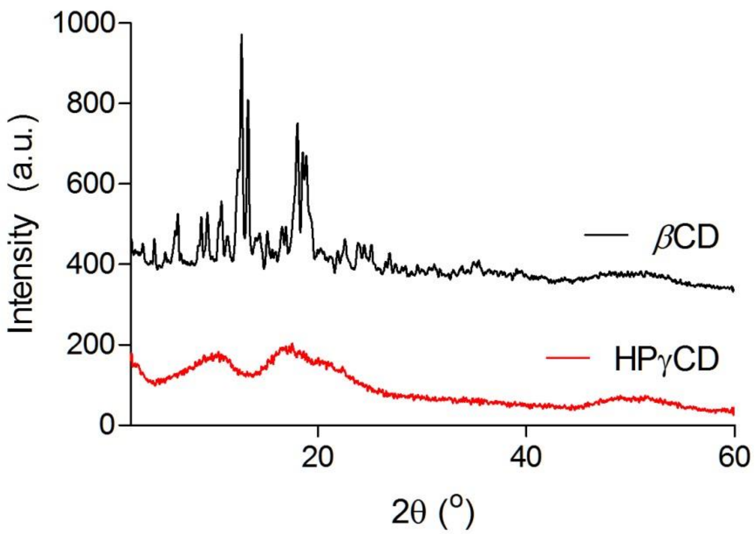

Figure S5: XRD patterns of as-received β-CD and HP-γ-CD.

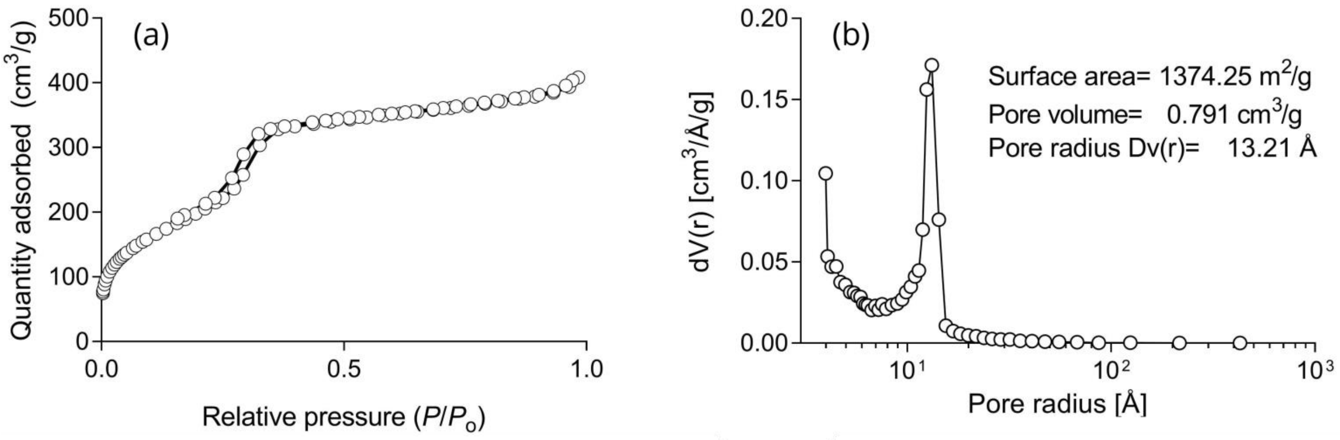

Figure S6: BET analyses for MSNs. (a) Nitrogen adsorption–desorption isotherm measured at 77 K and (b) BJH pore size analysis of the pristine MSNs (0.2% (w/v) CTAC) (MSN-20).

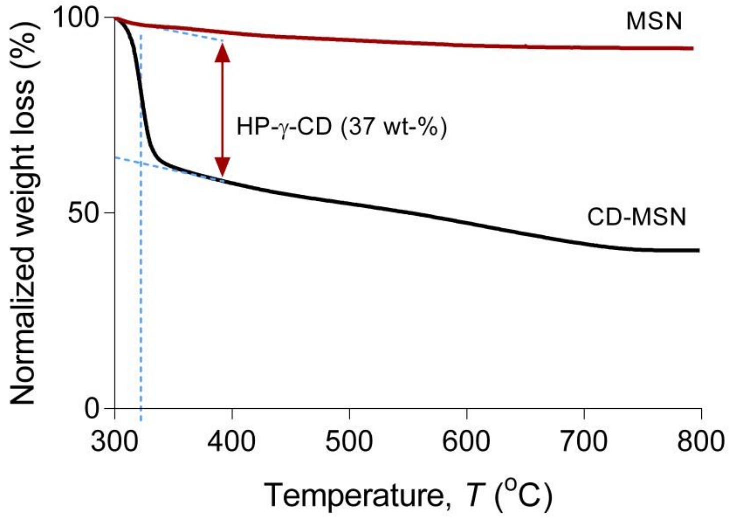

Figure S7: TGA curves of MSNs (MSN-9 and MSN-20) prepared in the presence and absence of CD. The dotted line corresponds to the burning of the embedded CD. c_{CTAC} = 0.2% (w/v) and $c_{HP\text{-}\gamma\text{-}CD}$ = 0.6% (w/v).

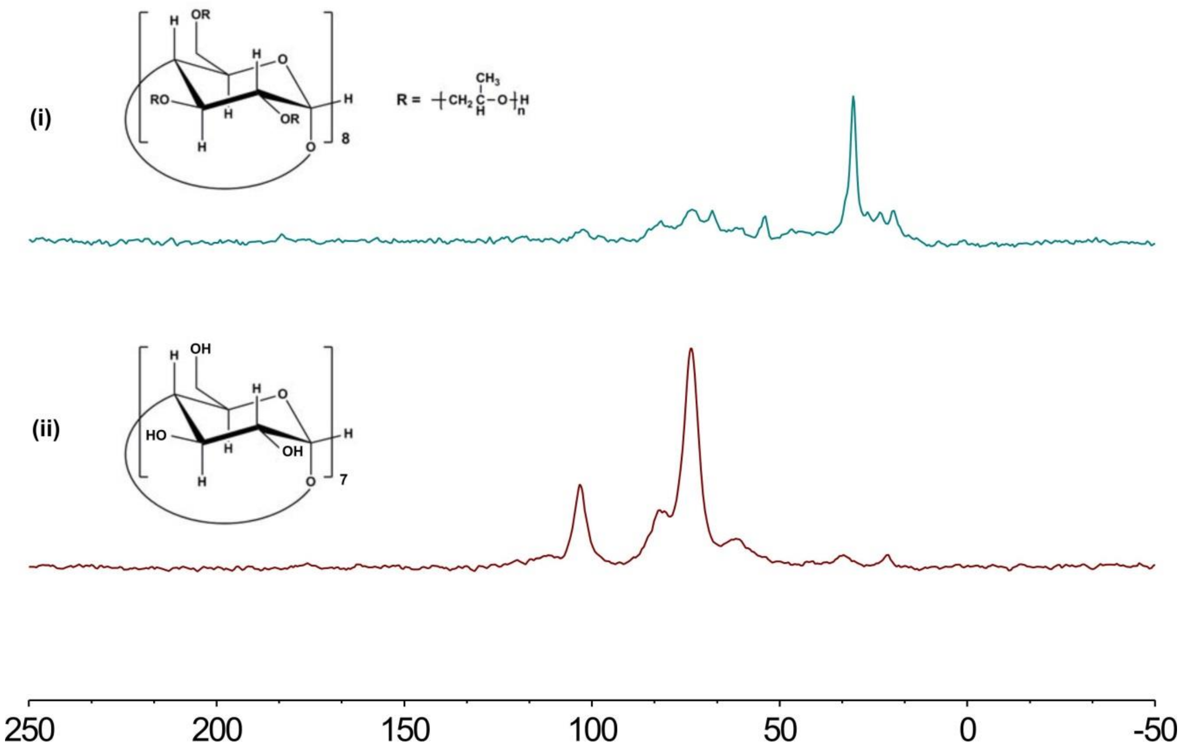

Figure S8: Solid-state ^{13}C NMR spectra of MSNs; (i) $c_{\text{HP-}\gamma\text{-CD}}$ = 0.25% (w/v) (MSN-3) and (ii) $c_{\beta\text{-CD}}$ = 1.5% (w/v) (MSN-15).
